# Selenium Nanoparticles Biosynthesized by *Pantoea agglomerans* and Their Effects on Cellular and Physiological Parameters in the Rainbow Trout *Oncorhynchus mykiss*

**DOI:** 10.3390/biology11030463

**Published:** 2022-03-17

**Authors:** Francisco Yanez-Lemus, Rubén Moraga, Luis Mercado, Carlos Jara-Gutierrez, Carlos T. Smith, Paulina Aguayo, Kimberly Sanchez-Alonzo, Apolinaria García-Cancino, Ariel Valenzuela, Victor L. Campos

**Affiliations:** 1 Environmental Microbiology Laboratory, Department of Microbiology, Faculty of Biological Sciences, Universidad de Concepcion, Concepcion 4070386, Chile; franciscoyanez@udec.cl (F.Y.-L.); csmith@udec.cl (C.T.S.); paulinaaguayo@udec.cl (P.A.); 2 Microbiology Laboratory, Faculty of Renewable Natural Resources, Arturo Prat University, Iquique 1100000, Chile; rmoraga@unap.cl; 3 Grupo de Marcadores Inmunológicos en Organismos Acuáticos, Laboratorio de Genética e Inmunología Molecular, Instituto de Biología, Pontificia Universidad Católica de Valparaíso, Valparaiso 2340000, Chile; luis.mercado@pucv.cl; 4 Centro de Investigaciones Biomédicas (CIB), Facultad de Medicina, Escuela de Kinesiología, Universidad de Valparaíso, Valparaiso 2340000, Chile; carlos.jara@uv.cl; 5 Faculty of Environmental Sciences, EULA-Chile, Universidad de Concepcion, Concepcion 4070386, Chile; 6 Institute of Natural Resources, Faculty of Veterinary Medicine and Agronomy, Universidad de Las Américas, Sede Concepcion, Chacabuco 539, Concepcion 3349001, Chile; 7 Laboratory of Bacterial Pathogenicity, Department of Microbiology, Faculty of Biological Sciences, Universidad de Concepcion, Concepcion 4070386, Chile; kimsanchez@udec.cl (K.S.-A.); apgarcia@udec.cl (A.G.-C.); 8 Laboratory of Pisciculture and Aquatic Pathology, Department of Oceanography, Faculty of Natural and Oceanographic Sciences, Universidad de Concepcion, Concepcion 4070386, Chile; avalenz@udec.cl

**Keywords:** Se nanoparticles, *Pantoea agglomerans*, selenite, rainbow trout, cell viability, antioxidant activity, food supplement, immune response, glutathione peroxidase, growth parameters

## Abstract

**Simple Summary:**

Nanoparticles (Nps), new biotechnological tools, possess unique physical and chemical properties and are increasingly being used in several fields, such as manufacture, medicine and veterinary medicine. In this work, we evaluated the effects of selenium (Se) nanoparticles stabilized with L-Cysteine (Se^0^Nps/L-Cys) as a nutritional supplement, to modulate immunological, oxidative status, and productive parameters in *O. mykiss.* The results demonstrated that Se^0^Nps/L-Cys showed less toxicity and higher antioxidant activity than Se^0^Nps and Na_2_SeO_3_. The Se^0^Nps/L-Cys, as a dietary supplement, had a significantly better effect on both immunological and physiological parameters, causing improvements at the productive level of *O. mykiss* when compared with Se^0^Nps and Na_2_SeO_3_. We concluded that Se^0^Nps sythetised by *P. agglomerans*, used as dietary supplement, is an environmentally friendly and promising alternative for nutritional supplementation for *O. mykiss*.

**Abstract:**

The applications of nanoparticles (Nps) as food additives, health enhancers, and antimicrobials in animal production are increasing. The aim of this study was to evaluate the effect of selenium (Se) nanoparticles (Se^0^Nps) stabilized with L-cysteine (Se^0^Nps/L-Cys), as a nutritional supplement, on immunological, oxidative status, and productive parameters in *O. mykiss.* TEM and SEM-EDS showed the accumulation of spherical Se^0^Nps entirely composed by elemental selenium (Se^0^) as intracellular and extracellular deposits in *Pantoea agglomerans* UC-32 strain. The in vitro antioxidant capacity of Se^0^Nps/L-Cys was significant more efficient ROS scavengers than Se^0^Nps and Na_2_SeO_3_. We also evaluate the effect of Se^0^Nps/L-Cys on cell viability and oxidative stress in RTgill-W1, RTS-11, or T-PHKM *Oncorhynchus mykiss* cell lines. Se^0^Nps/L-Cys showed less toxic and high antioxidant activity than Se^0^Nps and Na_2_SeO_3_. Finally, the dietary Se^0^Nps/L-Cys had a significant better effect on both plasma lysozyme and respiratory burst activity (innate immune response), on tissular Gpx activity (oxidative status), and on well-being (productive parameter) of *O. mykiss* when it is compared to Se^0^Nps and Na_2_SeO_3_. Se^0^Nps/L-Cys is a promising alternative for nutritional supplement for *O. mykiss* with better performance than Na_2_SeO_3_ and Se^0^Nps, ease to implementation, and reduced environmental impact.

## 1. Introduction

The rapid increase in the world population and its purchasing power explains the growing demand for food and the consequent rapid development of the aquaculture industry in recent decades [1,2]. Salmon farming is a relatively young industry, which harvested 230 thousand metric tons (mt) in 1990 and reached over 2 million mt in 2018 [3]. Globally, *Salmo salar* (Atlantic salmon) and *Oncorhynchus mykiss* (rainbow trout) are among the 15 most traded fish species [3]. The high animal density associated with aquaculture favors the appearance of chronic stress in fish, negatively affecting production [4]. In addition, in rainbow trout, chronic stress may induce oxidative stress (OE), [5] and organic depletion of vitamins and minerals, such as Se [6].

Se is an essential element for animals, and it participates in metabolic processes involved in development, growth, health, and fertility, and it is administered to cultured salmon as a nutritional supplement [7,8]. In addition, Se is a cofactor of multiple proteins (seleno-proteins), including glutathione peroxidase and thioredoxin reductase [9], enzymes which contribute to remove reactive oxygen species (ROS), preventing OE [10]. Kohshahi et al. [11], demonstrated the immune-stimulating effect of different Se chemical forms when included as a nutritional supplement to channel catfish (*Ictalurus punctatus*).

Hilton et al. [12] reported that the daily requirement of Se for rainbow trout is between 0.15 to 0.38 mg kg^−1^ dry-matter fed. Rider et al. [13] reported that, under stressful environmental conditions, the requirement could be increased up to 4.0 mg kg^−1^ (dry mass). Chronic consumption of 13 mg Se kg^−1^ (dry mass) caused evident signs of toxicity in rainbow trout, resulting, among others, in a decreased growth rate and high mortality [12].

Feeding fish, such as cultured salmonids, with high trophic levels requires the use of fishmeal and fish oil to adequately meet their nutritional needs [14]. Given the reduction in the stock of marine fish [8], food formulas are now including ingredients of vegetal origin to offset the fishmeal price increase [9]. According to Ytrestoyl et al. [14], multiple diets for salmonids include more than 70% of ingredients of plant origin.

Se natural concentration in fishmeal fluctuates between 1.5 and 3.1 mg kg^−1^ [15] while in vegetal ingredients it varies barely from 0.01 to 0.16 mg kg^−1^ [16]. Betancor et al. [17] reported that including raw material of vegetable origin to fishmeal could reduce the Se content in salmon fillet, reducing its nutritional value. This outcome may be the consequence of the presence of phytic acid in plants, reducing the availability of Se at the intestinal level of fish [18]. In order to achieve tissue concentrations of Se allowing an adequate development and well-being of farmed salmonid fish, it is necessary to supplement their diet with Se [19]. The chemical species of Se supplemented to fish, either organic (selenomethionine and selenocysteine) or inorganic (Na_2_SeO_3_), affects the bioavailability of the micronutrient and has an impact on their metabolism [20]. The inorganic form (Na_2_SeO_3_) is less bioavailable and has a greater toxicity than the organic Se species in rainbow trout [21].

Nanoparticles (Nps), new biotechnological tools, possess unique physical and chemical properties and are increasingly being used in several fields, such as imaging, chemical sensors and biosensors, diagnostics, drug delivery, catalysis, energy, photonics, medicine [22], and veterinary medicine [23]. The applications of Nps as food additives, health enhancers, and antimicrobials in animal production are increasing [24,25]. Several authors reported a higher bioavailability and lower toxicity of Se when administered as Nps (Se^0^Nps) when compared to other chemical forms of Se and also that dietary supplementation with Se^0^Nps in farmed fish contributes to the improvement of productive indices in intensive aquaculture [8,22,23,24,25,26].

Different chemical and physical methods have been described to produce Se^0^Nps. In general, these methods involve the use of toxic solvents, the generation of dangerous by-products, and high-energy consumption [27]. On the other hand, since they can grow rapidly and they are easy to manipulate and to culture at a relatively low cost, bacteria are being used as micro-factories capable of biosynthesizing metal Nps [28]. In addition, biogenic Nps, such as Se^0^Nps, can interact with different substances and the addition of functional chemical groups, or functionalization, (such as thiols, disulphurs, amines, carboxylic acids, phosphine, and other biomolecules) [29]. Functionalization provides Nps with advantages including, among others, inhibiting agglomeration, maintaining particle sizes compatible with metabolic activity, and improving bioavailability [29]. The above considerations encouraged us to produce and characterize functionalized Se^0^Nps (Se^0^Nps/L-Cys) and to evaluate if they showed better effects than non-functionalized Se^0^Nps or inorganic soluble Se (Na_2_SeO_3_) on cell viability and oxidative status in three types of cell cultures of *O. mykiss*. The effects of Se^0^Nps/L-Cys, Se^0^Nps, and Na_2_SeO_3_ as a nutritional supplement on immunological and oxidative status, and productive parameters for *O. mykiss* were also compared.

## 2. Materials and Methods

### 2.1. Biosynthesis, Purification, and Functionalization of Se^0^Nps

*Pantoea agglomerans* UC-32, isolated from the sediments of Camarones river, northern Chile, was reported as a bacterial strain capable to produce Se^0^Nps [30]. To produce Se^0^Nps, *P. agglomerans* UC-32—kept at the culture collection of the Laboratory of Environmental Microbiology, Department of Microbiology, Faculty of Biological Sciences, University of Concepcion, Concepcion, Chile—was cultured overnight under aerobic conditions in trypticase broth (TB) (Merck, Darmstadt, Germany) supplemented with 0.5 mM Na_2_SeO_3_ at 30 °C with agitation (100 rpm) [30]. Cultures without Na_2_SeO_3_ were used as negative control. The purification of Se^0^Nps biosynthesized by *P. agglomerans* UC-32 and its functionalization with L-cysteine were done as described by Chen et al. [25] and Tarrahi et al. [31], respectively. L-cysteine functionalized Se^0^Nps (Se^0^Nps/L-Cys) were resuspended in 10 mL Leibovitz’s L-15 medium (Gibco, Waltham, MA, USA) and stored at −80 °C. Non-functionalized Se^0^Nps were obtained from fresh culture and stored at −80 °C.

### 2.2. Characterization of Se^0^Nps Biosynthesized by P. agglomerans UC-32 Strain

The morphology and size of *P. agglomerans* UC-32 biosynthesized Se^0^Nps and Se^0^Nps/L-Cys were evaluated by means of transmission electron microscopy (TEM) as described by Dhanjal and Cameotra [32] using a JEOL JSM 1200EX-II TEM microscope (JEOL, Peabody, MA, USA). Their chemical characterization was done by means of scanning electron microscopy-energy dispersive X-ray Spectroscopy (SEM-EDS), as described by Torres et al. [30], using a JEOL JSM 6380LV SEM microscope (JEOL, Peabody, MA, USA).

### 2.3. Antioxidant Capacity of Se^0^Nps/L-Cys

The antioxidant capacity of Se^0^Nps/L-Cys, Se^0^Nps, and Na_2_SeO_3_ was measured on the basis of their scavenging ROS capacity using three assays: the radical scavenging 2,2′-diphenyl-1-picrylhydrazyl (DPPH) assay, the ferric reducing antioxidant power (FRAP) assay, and the total radical-trapping antioxidant parameter assay (TRAP). The DPPH assay was done following the procedure of Brand-Williams et al. [33]. The IC_50_ value was calculated to determine the concentration of the sample required to inhibit 50% of the radicals. The lower the IC_50_ value, the higher the antioxidant activity of samples [34]. The FRAP assay was done as described by Dudonné et al. [35] and the absorbance values obtained were interpolated in a Trolox calibration curve (0–200 mg L^−1^). The TRAP assay was done according to Romay et al. [36], and the absorbance values were interpolated in a Trolox standard curve (0–120 mg L^−1^). The absorbances of all three assays were obtained using an Epoch model microplate spectrophotometer (BioTek Instruments, Inc., Winooski, VT, USA) adjusted to the appropriate wavelength for each assay. DPPH values were expressed as half-maximal inhibitory concentration (IC_50_) in mg mL^−1^. FRAP and TRAP values were expressed in mM Trolox equivalent antioxidant capacity (mM TEAC). Vitamin C (Merck, Darmstadt, Germany), Trolox (Merck, Darmstadt, Germany), and N-acetylcysteine (NAC) (Merck, Darmstadt, Germany) were used as positive controls. Different concentrations of Se^0^Nps/L-Cys, Se^0^Nps, or Na_2_SeO_3_, in the range of 50–500 μg mL^−1^ in methanolic solution, were added to DPPH, FRAP, or TRAP solutions.

### 2.4. Effect of Se^0^Nps/L-Cys in Rainbow Trout’s Cells Culture (In Vitro Model)

#### 2.4.1. Oncorhynchus Mykiss Cell Lines and Primary Head Kidney Monocyte-like Cells Culture

For in vitro assays, *O. mykiss* cell lines RTgill-W1 (normal epithelial gill cells; ATCC -CRL2523) and RTS-11 (spleen, monocyte/macrophage-like cells; RRID:CVCL_F835) and primary head kidney monocyte-like (T-PHKM) culture cells were provided by Dr. Luis Mercado (Pontifical Catholic University of Valparaíso, Valparaíso, Chile). RTgill-W1 and RTS-11 cells were cultured in Leibovitz’s L-15 medium (Gibco, Waltham, MA, USA) supplemented with 2% penicillin streptomycin (100 mg mL^−1^ streptomycin, 100 IU mL^−1^ penicillin (Gibco, Waltham, MA, USA) and 10% fetal calf serum (FCS) (Gibco, Waltham, MA, USA) for RTgill-W1 cells or 30% FCS for RTS-11 cells. T-PHKM cells were obtained and cultured according to Abarca et al. [37]. The three cell lines were stabilized at 18 °C overnight before been exposed to Se^0^Nps/L-Cys or Na_2_SeO_3_.

#### 2.4.2. In Vitro Analysis of the Toxicity of Se^0^Nps/L-Cys

RTgill-W1 cells (4 × 10^4^), RTS-11 cells (5 × 10^4^) or T-PHKM cells (5 × 10^4^) in 100 µL Leibovitz’s L-15 medium were placed in each well of 96 wells flat bottom microplates (Merck, Darmstadt, Germany) and cultured at 20 °C. After 18 h of incubation, the culture medium was replaced with fresh medium supplemented with FCS and antibiotics, as indicated above, plus 160, 320, or 640 nM of Se^0^Nps/L-Cys or Na_2_SeO_3_. The stock Se^0^Nps/L-Cys suspension or selenite solution were prepared in L-15 Leibovitz’s medium. Based on the data reported by Torres et al. [30], three concentrations of either Se^0^Nps/L-Cys or Na_2_SeO_3_ (160, 320, or 640 nM) were used. L-15 Leibovitz’s medium plus RTgill-W1, RTS-11, or T-PHKM cells was used as control in every experiment. After 23 h of culture, 10 µL of 2-(4-iodophenyl)-3-(4-nitrophenyl)-5-(2,4-disulfophenyl)-2H—tetrazolium monosodium salt (WST-1) (Roche Applied Science, Indianapolis, IN, USA) were added to each well following the manufacturer’s instructions. Cellular viability was measured at 450 nm using an Epoch microplate spectrophotometer (BioTek Instruments, Inc., Winooski, VT, USA). Cytotoxicity of Se^0^Nps/L-Cys or Na_2_SeO_3_ was expressed as percentage of viable cells with respect to the control. All experiments were carried out in triplicate.

#### 2.4.3. In Vitro Effect of Se^0^Nps/L-Cys against H_2_O_2_-Induced Oxidative Stress on Rainbow Trout Cell Cultures 

The effect of Se^0^Nps/L-Cys against H_2_O_2_-induced toxicity was evaluated in RTgill-W1, RTS11, and T-PHKM cells measuring cellular ROS concentration according to Singh et al. [38]. Briefly, RTgill-W1 cells (4 × 10^4^ cells), RTS-11 cells (5 × 10^4^ cells) or T-PHKM cells (5 × 10^4^ cells) in 100 µL L-15 Leibovitz’s medium were placed in each well of 96-well flat bottom microplates (Merck, Darmstadt, Germany) and cultured at 20 °C with Se^0^Nps/L-Cys or Na_2_SeO_3_ (160, 320 or 640 nM) for 24 h. Then, L-15 Leibovitz’s medium was carefully extracted and replaced with fresh medium containing 100, 150, or 300 μM hydrogen peroxide (H_2_O_2_) as a cellular ROS-inducing agent [39] at 20 °C during 24 h. According to Kling and Olsson [40], H_2_O_2_ concentrations chosen were non-lethal for rainbow trout cell lines. After this incubation period, 1 µM of the fluorescent probe 6-carboxy-2′,7′-diclorodihidrofluroresceine diacetate (Carboxy-DCFH-DA) (Molecular Probes/Invitrogen, Waltham, MA, USA) was added and cultures maintained at 20 °C in the dark for additional 30 min. The oxidation of carboxy-DCFH into highly fluorescent 2′,7′-dichlorofluorescein (DCF) by intracellular ROS was evaluated by the fluorescent absorbance value using a microplate reader PR 4100 TSC (Bio-Rad, Hercules, CA, USA). Cells were sampled and fluorescence was measured according to Chen et al. [41]. The ROS effects on cell viability of RTgill-W1, RTS11, and T-PHKM cells were also determined using the same procedure described in Section 2.4.2. The assays were carried out in triplicate.

### 2.5. Effect of Se^0^Nps/L-Cys Supplemented Food in Rainbow Trout’s (In Vivo Model)

#### 2.5.1. Feeding Trial Design

All animals used in this study were treated in accordance with the Biosecurity Regulations and Ethical Protocols approved by University of Concepcion Ethics Committee. Apparently healthy 160 rainbow trout having an initial weight of 104.53 ± 8.47 g (mean ± SE) and an initial length of 20.8 ± 3.32 cm (mean ± SE) were obtained from Salmones Pangue (Florida, Chile) and transported to an environmentally controlled semi-closed recirculation system (Laboratory of Pisciculture and Aquatic Pathology (LPAP)), Faculty of Natural Sciences and Oceanography, University of Concepcion, Concepcion, Chile. Trout were kept in fiberglass tanks, at 15.5 ± 0.8 °C and a maximum density of 25 kg fish m^−3^, containing 8.1 ± 0.08 mg L^−1^ dissolved oxygen and under a 12:12 light:dark photoperiod [42]. Twenty fish were randomly distributed in each one of 8 tanks. Two tanks were assigned to each one of the below described four different diets assayed, totalling 40 fish per diet group. Fish were acclimated for 21 days, time span in which they were fed an acclimatization diet including the minimum rainbow trout selenium requirement according to the National Research Council (NRC) [19]. The four diets were administered during a 30-day period; one group (control group) received the same acclimatization diet. The three experimental diets were enriched with 5 mg of Se nanoparticles (Se^0^Nps), of L-cysteine functionalized Se nanoparticles (Se^0^Nps/L-Cys), or inorganic Se (Na_2_SeO_3_) per kg dry food to obtain a non-toxic diet [13,14]. To prepare the diets, the approximate yield of Se^0^Nps of a 1 L culture of *P. agglomerans* culture was determined. All diets were prepared weekly, according to Vera [43], by Cargill-Ewos (Coronel, Chile) containing 39–43% crude protein, 10–16% lipid, 3–4% fiber, 9–12% ash, 7–13% moisture, 1–2% calcium, and 1–1.4% phosphate. Fish were fed twice daily (10:00 h and 16:00 h) receiving 2% of their average body weight per day. Eight fish per tank were weighed (BLC 1500 scale, Boeco, Hamburg, Germany) at the beginning of the feeding trial, and subsequently when samples were taken, and the amount of food given adjusted accordingly.

#### 2.5.2. Fish Sampling

On days 0, 15, and 30, six fish from each experimental or control group were carefully captured, sacrificed by an overdose of the anesthetic BZ-20 (50 ppm of sodium pa-ra-aminobenzoate in fresh water; Veterquimica, Santiago, Chile) and then individually weighted (BLC 1500 scale; Boeco, Hamburg, Germany) and measured from the tip of the snout to the rear edge of the fork at the center of the tail fin. Blood was extracted from the caudal vein of each fish, by means of a heparinized 18G needle fitted to a 5 mL syringe and transferred to sterile microtubes containing 0.02 mL of 1000 U mL^−1^ heparin (Merck, Darm-stadt, Germany). Additionally, samples of liver and dorsal muscle were obtained. Samples were immediately transported at 4 °C to the Laboratory of Environmental Microbiology, University of Concepcion, where plasma was obtained by centrifugation at 5000× *g* for 10 min, and liver and dorsal muscle were fragmented. Then, plasma, liver and dorsal muscle were stored at −80 °C.

#### 2.5.3. Innate Immune Responses

Plasma lysozyme activity and ROS concentration in white blood cells (WBCs) of six rainbow trout per sampling day and diet were measured. A turbidimetric assay was used to determine plasma lysozyme activity level [44]. Briefly, 950 µL of buffered substrate (0.25 mg of *Micrococcus lysodeikticus* in 1 mL of buffered 40 mM sodium phosphate pH 6.2) was mixed with 50 µL of fish plasma. The absorbance of the samples was measured at times 0 and 30 min of incubation at room temperature by means of an Epoch spectrophotometer at 450 nm (BioTek Instruments, Inc., Winooski, VT, USA). A 0.001 min^−1^ absorbance reduction was evaluated as one unit of lysozyme activity [44].

For ROS concentration measurements, an assay evaluating the reduction of nitroblue tetrazolium (NBT) into colored formazan by oxidizing agents was used following the method of Anderson and Siwicki [45].

#### 2.5.4. Activity of the Antioxidant Enzyme Glutathione Peroxidase (Gpx)

The glutathione peroxidase (Gpx) activity was assayed in plasma, according to Lawrence and Burk [46] and liver and dorsal muscle as described by Fontagné-Dicharry et al. [21]. Gpx activity in plasma samples was evaluated immediately after thawing. In the case of liver and muscle, samples were rapidly thawed and homogenized in 10 volumes (w/v) of ice-cold saline for 3 min and centrifuged for 15 min at 4000× *g* and the supernatants collected to evaluate the activity of GPx. Gpx activity present in the supernatants was measured in a solution of 50 mM phosphate buffer (pH 7.4), 1 mM EDTA (Merck, Darmstadt, Germany), 2 mM sodium azide (Merck, Darmstadt, Germany), 2 mM reduced glutathione (GSH) (Merck, Darmstadt, Germany), 0.1 mM NADPH (Merck, Darmstadt, Germany), and 0.2 mM glutathione reductase (Merck, Darmstadt, Germany) following the reduction of H_2_O_2_ (50μM) at 30 °C and 340 nm. One unit of Gpx activity was reported as l mol NADPH consumed per min per mg of plasma protein, using the appropriate molar absorptivity coefficient for NADPH (6220 mol L^−1^ cm^−1^). Plasma proteins were measured by the method of Lowry et al. [47].

#### 2.5.5. Effect of Se^0^Nps/L-Cys on Trout Growth Performance and Survival Rate

The effects of Se^0^Nps, Se^0^Nps/L-Cys or Na_2_SeO_3_ on productive parameters of the fish were evaluated every five days. Weight and length of each trout and the number of dead fish were recorded to calculate the specific growth rate (SGR), weight gain (WG), condition factor (CF), and survival rate (%), according to Naderi et al. [8] and Lugert et al. [48] using the following Equations (1)–(4):

(1) $$\mathrm{SGR}\;\left(\%\;\mathrm{increase}\;\mathrm{body}\;\mathrm{wt}\;d.^{-1}\right)=\left[\frac{\left(\ln w2-\ln w1\right)}{\mathrm{days}}\right]\times 100$$

(2) WG (g)=w2−w1

(3) $$\mathrm{CF}\;=\left[\frac{w}{L^{3}}\right]\times 100\;$$

(4) $$\mathrm{Survival}\;\mathrm{rate}\;\left(\%\right)=\left[\frac{n2}{n1}\right]\times 100$$

where w1 = starting weight (g); w2 = final weight (g); days = days in the growth period; w = weight (g); L = length (cm); n1 = initial number of fish; n2 = final number of fish.

When the three different diet and one-control groups were made up, the initial condition factor (ICF) was considered (similar sizes and weights) to make sure that the initial populations of each group were homogeneous with respect to the development stage and the nutritional condition.

### 2.6. Statistics

One-way analysis of variance (ANOVA) followed by an LSD multiple comparison test was used to determine the statistical significance for multiple comparisons. The Student’s *t*-test was used for pairwise comparisons. Values of *p* < 0.05 were considered as statistically significant. All statistical tests were performed using the GraphPad Prism software version 7 for Windows (GraphPad Software, San Diego, CA, USA, www.graphpad.com, accessed on 22 July 2020).

## 3. Results

### 3.1. Characterization of Se^0^Nps Biosynthesized by P. agglomerans UC-32 without and after Functionalization

**Figure 1:**
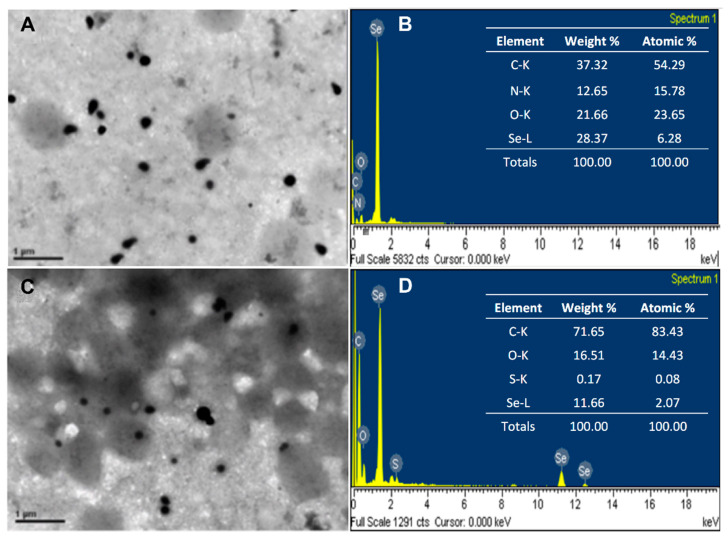
Selenium nanoparticles produced by *Pantoea agglomerans* UC-32 strain without functionalization (Se^0^Nps) and after L-cysteine functionalization (Se^0^Nps/L-Cys). (**A**) TEM micrograph of Se^0^Nps among *P. agglomerans* UC-32 cell debris; (**B**) SEM-EDS data obtained from Se^0^Nps; (**C**) TEM micrograph of Se^0^Nps/L-Cys among *P. agglomerans* UC-32 cell debris; (**D**) SEM-EDS data obtained from Se^0^Nps/L-Cys.

The size and morphology of the biosynthesized Se^0^Nps and Se^0^Nps/L-Cys were analyzed by TEM. TEM observations revealed that both Se^0^Nps and Se^0^Nps/L-Cys were sphere-like nanoparticles with sizes between 53 to 170 nm and 32 to 160 nm in diameter, respectively (Figure 1A,C, respectively), which indicated that Se^0^Nps/L-Cys were significantly smaller than non-functionalized Se^0^Nps (*p* < 0.05). SEM-EDS analysis of Se^0^Nps and Se^0^Nps/L-Cys showed the presence of peaks corresponding to Se, confirming that the nanoparticles were mainly composed of Se. The presence of C, N, and O signals can be ascribed to cell debris (Figure 1B,D). In the case of Se^0^Nps/L-Cys, a sulfur (S) peak was, as expected, also observed due to the thiol sidechain of cysteine, confirming their functionalization (Figure 1D).

### 3.2. Antioxidant Capacity of Se^0^Nps/L-Cys

The antioxidant activity of Se^0^Nps/L-Cys, Se^0^Nps and Na_2_SeO_3_ was evaluated in vitro using the DPPH, FRAP, and TRAP assays (Table 1). Data in Table 1 were obtained by using 500 µg mL^−1^ of Se^0^Nps/L-Cys, Se^0^Nps, or Na_2_SeO_3_.

The DPPH assay showed that the ROS scavenger activity of the positive controls (Vit C, Trolox, and NAC) was significantly better than that of the three forms of Se tested. Se^0^Nps/L-Cys and Se^0^Nps were more efficient ROS scavengers than Na_2_SeO_3_ (*p* < 0.05). When comparing both types of Nps, the functionalized ones were significantly better (*p* < 0.05) ROS scavengers than the non-functionalized ones.

Regarding the FRAP assay, the positive Vit C control was a better ROS scavenger than the NAC control and all three Se compounds (*p* < 0.05). Antioxidant capacity of Se^0^Nps was higher than Se^0^Nps/L-Cys (*p* > 0.05) and Na_2_SeO_3_ (*p* < 0.05). Finally, the TRAP assay showed that Vit C control had the highest antioxidant activity (*p* < 0.05). Regarding Se compounds, a similar antioxidant activity pattern to DPPH was detected. Se^0^Nps/L-Cys was a significant better ROS scavenger than Se^0^Nps and Na_2_SeO_3_ (*p* < 0.05).

### 3.3. Toxicity of Se^0^Nps/L-Cys for Cell Lines RTgill-W1 and RTS-11 and Primary Culture T-PHKM

The toxicity of Se^0^Nps/L-Cys for the cells was expressed in percentage of viable RTgill-W1, RTS-11, or T-PHKM cells when co-cultured with Se^0^Nps/L-Cys or, for comparison, Na_2_SeO_3_ during 24 h (Table 2). The cytotoxicity for both cell lines and the primary culture was dose dependent showing a decreasing cell viability as the concentration of Se^0^Nps/L-Cys or Na_2_SeO_3_ increased. When comparing with the control, the viability of the cells assayed was not significantly reduced (*p* < 0.05) only when RTgill-W1 (95.64%), RTS-11 (96.39%) or when T-PHKM (96.52%) cells were exposed to either 160 nM Se^0^Nps/L-Cys or 160 nM Na_2_SeO_3_. When comparing the effect of a same Se^0^Nps/L-Cys or Na_2_SeO_3_ concentration, all three cell types showed higher viabilities when exposed to 160, 320, or 640 nM Se^0^Nps/L-Cys than to Na_2_SeO_3_. Results for RTgill-W1 cells showed significant higher viabilities (*p* < 0.05) when they were exposed to 160, 320, or 640 nM Se^0^Nps/L-Cys than to Na_2_SeO_3_. In the case of RTS-11 cells, viabilities when exposed to 640 nM Se^0^Nps/L-Cys or Na_2_SeO_3_ were 95.67% and 93.74%, respectively, when compared to the control (*p* < 0.05). On the other hand, similar concentrations of Se^0^Nps/L-Cys or Na_2_SeO_3_ caused no significant differences (*p* > 0.05) in the survival of T-PHKM cells. Finally, the analysis of cell viability at different concentrations of the same form of Se (Se^0^Nps/L-Cys or Na_2_SeO_3_) showed significant differences (*p* < 0.05) between 160 nM and 640 nM in the three cellular types, being 640 mM more toxic than 160 mM of both Se sources.

### 3.4. Effect of Se^0^Nps/L-Cys on H_2_O_2_-Induced Oxidative Stress in Cell Lines RTgill-W1 and RTS-11 and T-PHKM Primary Cell Culture

**Figure 2:**
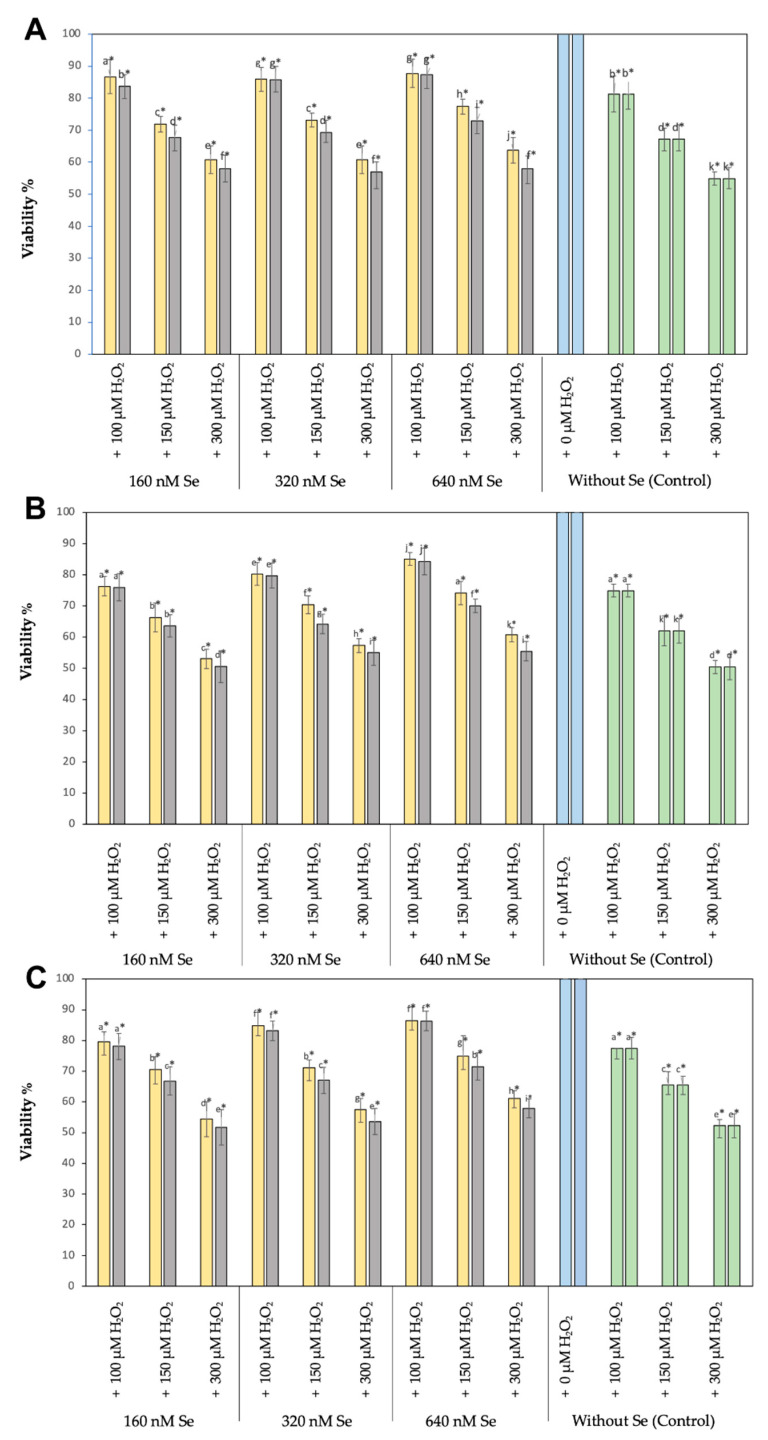
Cell viability (as percentage of the negative control 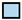) of cell lines RTS-11 (**A**), RTgill-W1 (**B**), and of the primary culture T-PHKM (**C**) treated with Se^0^Nps/L-Cys 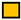 or Na_2_SeO_3_ 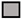 and then subjected to H_2_O_2_ as a ROS inducing agent. All data is given as mean ± SD. Positive controls 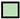. Different letters on top of bars indicate significant differences among groups. * Statistically different from the negative control.

A significant reduction (*p* < 0.05) in cell viability was observed when the cell viability of all three cell types treated with 100, 150, or 300 µM H_2_O_2_ (positive controls) was compared to cells not treated with H_2_O_2_ (negative controls), being the highest H_2_O_2_ concentration the one causing the largest cell viability reduction in the three cell lines assayed. When the cell viability of the three cell types was compared, RTS cells demonstrated better viabilities than RTgill-W1 or T-PHKM cells when subjected to 100, 150, or 300 µM H_2_O_2_ (*p* < 0.05) (Figure 2).

RTS-11, RTgill-W1, and T-PHKM cells cultured in the presence of 160, 320, or 640 nM Se^0^Nps/L-Cys or Na_2_SeO_3_ and then subjected to 100, 150, or 300 µM H_2_O_2_ showed to better retain their viability when compared to the positive controls in a dose dependent manner, being the best cell viabilities obtained in the cultures containing 640 nM Se^0^Nps/L-Cys or Na_2_SeO_3_. Similarly, as observed in the positive controls, RTS-11 cells showed better viabilities when compared to RTgill-W1 or T-PHKM at all Se^0^Nps/L-Cys or Na_2_SeO_3_ concentrations. RT-gill-W1 was the cell type showing the lowest cell viabilities. Cell viabilities in all experimental groups were significantly less (*p* < 0.05) than those of the negative controls and significantly better (*p* < 0.05) than those of positive controls (Figure 2).

**Figure 3:**
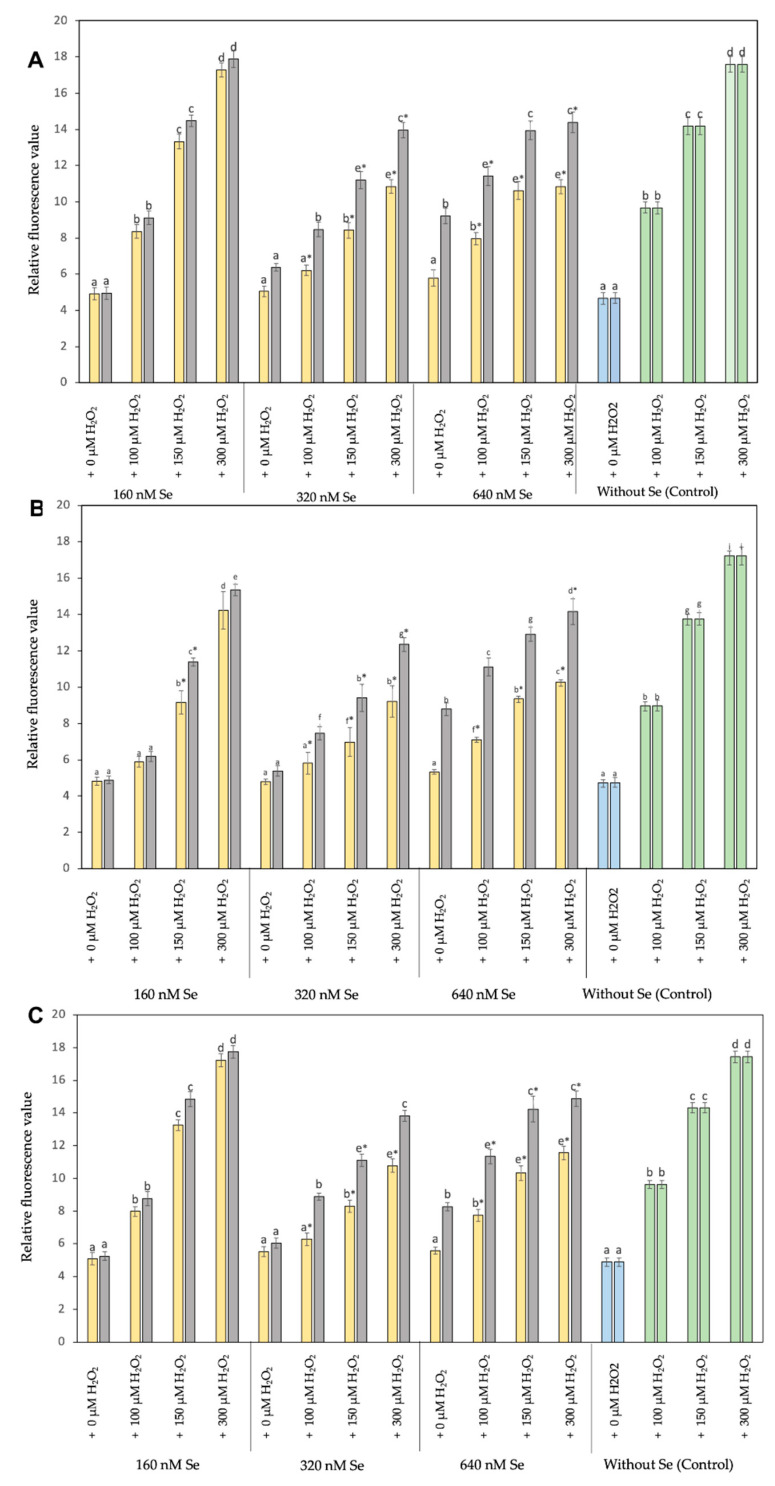
Level of intracellular ROS on cell lines RTS-11 (**A**), RTgill-W1 (**B**), and of the primary culture T-PHKM (**C**) treated with Se^0^Nps/L-Cys 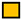 or Na_2_SeO_3_ 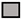 and then subjected to H_2_O_2_ as a ROS inducing agent. All data is given as mean ± SD. Different letters on top of bars indicate significant differences among groups. * Significant reduction of cellular ROS concentration compared to the positive controls 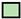. Negative control 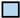.

Se^0^Nps/L-Cys (Figure 2) showed to provide a better protection than Na_2_SeO_3_ to RTS-11, RTgill-W1, and T-PHKM cells in all the experimental groups exposed to H_2_O_2_ (Figure 3). RTS-11 cells cultured in the presence of Se^0^Nps/L-Cys showed to retain a better cell viability than the one achieved in the presence of Na_2_SeO_3_, being it significant in the experimental groups 160 + 100 (86.67% vs. 83.61%, respectively), 160 + 150 (71.85% vs. 67.69%, respectively), 160 + 300 (60.74% vs. 57.99%, respectively), 320 + 150 (73.16% vs. 69.28%, respectively), 320 + 300 (60.81% vs. 57.04%, respectively), 640 + 150 (77.39% vs. 72.93%, respectively), and 640 + 300 (63.76% vs. 58.05%, respectively). On the other hand, RTgill-W1 cells plus Se^0^Nps/L-Cys showed a significant better viability that the same cell type plus Na_2_SeO_3_ in the experimental groups 160 + 300 (53.01% vs. 50.55%, respectively), 320 + 150 (70.37% vs. 64.16%, respectively), 640 + 150 (74.11% vs. 70.02%, respectively), 320 + 300 (57.32% vs. 54.98%, respectively), and 640 + 300 (60.81% vs. 55.45%, respectively). Finally, primary culture T-PHKM cells subjected to Se^0^Nps/L-Cys showed a viability significantly better than T-PHKM subjected to Na_2_SeO_3_ in the groups 160 + 150 (70.51% vs. 66.82%, respectively), 160 + 300 (54.40% vs. 51.73%, respectively), 320 + 150 (71.13% vs. 67.03%, respectively), 320 + 300 (57.61% vs. 53.63%, respectively), 640 + 150 (74.88% vs. 71.49%, respectively), and 640 + 300 (61.14% vs. 57.91%, respectively) (Figure 2).

### 3.5. In Vitro Effect of Se^0^Nps/L-Cys on ROS Concentration in Cell Lines RTgill-W1 and RTS-11 and Primary Culture T-PHKM

The effect of Se^0^Nps/L-Cys on ROS scavenging was evaluated on RTgill-W1, RTS-11, and T-PHKM cells pre-treated with Se^0^Nps/L-Cys or Na_2_SeO_3_ and then subjected to H_2_O_2_. The assay used measures the fluorescence emitted by DCF resulting from the oxidation of carboxy-DCFH by intracellular ROS.

As shown in Figure 3, the concentration of cellular ROS in RTgill-W1, RTS-11, and T-PHKM cells co-cultured with Se^0^Nps/L-Cys or Na_2_SeO_3_ (160, 320, or 640 nM) for 24 h was increased respect to each cell type negative control (only cells) in a concentration-dependent manner. T-PHKM cells co-cultured with 320 nM Na_2_SeO_3_ and RTgill-W1, RTS-11, and T-PHKM cells co-cultured with 640 nM Na_2_SeO_3_ significantly increased the cellular ROS concentration compared to observed in the negative control and three cell types under similar concentration of Se^0^Nps/L-Cys (*p* < 0.05).

A markedly increased (*p* < 0.05) cellular ROS of H_2_O_2_-induced RTgill-W1, RTS-11, and T-PHKM positive controls cells (cells plus 100, 150, or 300 µM H_2_O_2_) in a dose-dependent manner when compared to the respective negative controls, was observed. RTgill-W1, RTS-11, and T-PHKM cells pre-incubated with 160, 320, or 640 nM Se^0^Nps/L-Cys significantly reduced the increased cellular ROS concentration induced by 100, 150, or 300 µM H_2_O_2_ in a concentration-dependent manner compared to registered in positive controls (Figure 3). A better performance in reducing cellular ROS concentration of Se^0^Nps/L-Cys than Na_2_SeO_3_ in all experimental groups of each cell type, was noted (Figure 3). Se^0^Nps/L-Cys was a significant (*p* < 0.05) better cellular ROS concentration reducer than Na_2_SeO_3_ in 320 + 100, 320 + 150, 320 + 300, 640 + 100, 640 + 150, and 640 + 300 groups in RTgill-W1, RTS-11, and T-PHKM cells.

### 3.6. Effect of Se^0^Nps/L-Cys Supplemented Food in Rainbow Trout (In Vivo Model)

#### 3.6.1. Innate Immune Responses

Plasma lysozyme activity was assessed by its capacity to lyse *Micrococcus lysodeikticus* and ROS production by leukocytes was assessed by an assay evaluating NBT reduction. Regarding plasma lysozyme activity (Table 3). Since day 15, the plasma lysozyme activity of fish receiving Se^0^Nps/L-Cys supplemented food was significantly increased when compared to the control group and the group receiving Na_2_SeO_3_ supplement food (*p* > 0.05). On day 30, lysosome activity of the group receiving Se^0^Nps/L-Cys supplemented food was also significantly higher than the group receiving Se^0^Nps supplemented food (*p* > 0.05).

ROS production by peripheral leukocytes was evaluated by the reduction of NBT into the colored compound formazan; therefore, higher absorbances at the wavelength at which formazan absorbs correspond to higher ROS concentrations (Table 4). On the day 15, an increase of cellular ROS of trout receiving Se^0^Nps (*p* < 0.05) or Se^0^Nps/L-Cys (*p* > 0.05) when compared to the control group, was observed. Samplings on day 30 showed that the group receiving Se^0^Nps/L-Cys supplemented food significantly increased formazan levels, when compared to the groups whose diet was supplemented with Se^0^Nps or Na_2_SeO_3_ (*p* < 0.05). On day 30, ROS concentration in the group receiving Se^0^Nps was also significantly higher than the one in the group receiving Na_2_SeO_3_ (*p* < 0.05).

#### 3.6.2. Activity of the Antioxidant Enzyme Gpx

The activity of the enzyme Gpx in plasma, liver, and dorsal muscle of rainbow trout fed with Se^0^Nps, Se^0^Nps/L-Cys, or Na_2_SeO_3_ supplemented food for 30 days is shown in Table 5. Significant increases in Gpx activity were observed in plasma, liver, and dorsal muscle in the three groups receiving Se supplemented diet when compared to the control group (*p* < 0.05). Moreover, the group receiving the diet supplemented with Se^0^Nps/L-Cys showed a significant higher muscle tissue Gpx activity when compared to the group receiving Se^0^Nps (*p* < 0.05) and a significant higher Gpx activity in plasma, liver, and muscle tissue when compared to the group receiving Na_2_SeO_3_ (*p* < 0.05).

#### 3.6.3. Growth Performance and Survival

Growth performance and survival rate of fish receiving the different dietary treatments during the 30 days of analysis is shown in Table 6. Weight gain (WG) and specific growth rate (SGR) values were not significantly different among groups (*p* > 0.05). Nevertheless, the final condition factor (FCF) of trout fed food enriched with Se^0^Nps/L-Cys (1.68%) was significantly higher than FCF of the control group (1.27%), Se^0^Nps (1.52%), and Na_2_SeO_3_ (1.45%) groups (*p* < 0.05).

## 4. Discussion

Se is an essential element used by animal organisms, including fish, to carry out physiological processes for an adequate development as required by each species [19]. This chemical element indirectly contributes to remove and prevent oxidative stress, acting as an exogenous antioxidant [48], and plays an integral role in the immune and endocrine systems [49].

Intense fish culture systems maximize the effect of stressors, favoring the rising of diseases along with ensuing important economic losses [50]. According to Baldissera et al. [51], the onset and progression of fish infectious diseases are usually mediated by oxidative stress as well as oxidative damage. Thus, the supplementation of salmonid fish food with Se is necessary to maintain the optimal health and growth of farm-raised fish [52]. Nevertheless, there are conflicting reports on the literature about the effects of different sources of Se supplementation, including inorganic Se and Se nanoparticles, on the physiological parameters of fish species [53].

In the present work, predominantly spherical Se^0^Nps were produced by the cytoplasmic Na_2_SeO_3_ reduction by the bacterium *P. agglomerans* [54]. The detection of a sulfur (S) peak by SEM-EDS only in L-Cys treated Se^0^Nps (Se^0^Nps/L-Cys) confirmed the functionalization of the nanoparticles. L-Cys has proven to be effective as a functionalizing agent for nanoparticles due to the presence of a SH group in its structure [30]. According to Prasanth and Sudarsanakumar [55], Se^0^Nps functionalization with L-Cys results from the anchoring of the thiol group of cysteine to the surface of the nanoparticles.

TEM results showed that Se^0^Nps/L-Cys were significantly smaller than non-functionalized Se^0^Nps. This phenomenon could be associated to the anti-agglomeration property of L-Cys as reported by Perni et al. [56], who indicated L-Cys reduces the surface energy of the silver (Ag) nanoparticles enhancing their separation and preventing further agglomeration. L-Cys has been used as a functionalizing agent not only for Se^0^Nps but also for other Nps of other chemical composition, such as copper (Cu) [57], zinc (Zn) [58], silver (Ag) [59], and gold (Au) [60]. Several authors have reported the use of L-Cys as a functionalizing agent to obtain smaller Nps [35,61,62].

Our results suggest that Se^0^Nps/L-Cys were more effective as in vitro ROS scavengers than Na_2_SeO_3_. The higher ROS scavenging activity of the functionalized Nps, when compared to non-functionalized Se^0^Nps, also suggests that the smaller size of the functionalized SeNps and the independent ROS scavenging activity of L-Cys anchored to the surface of the Se^0^Nps/L-Cys combine their effects to increase the ROS scavenging activity exerted by Se^0^Nps/L-Cys. With respect the involvement of the size of the Nps on their ROS scavenging capacity, Huang et al. [63] concluded that the ROS scavenging effect, measured by the DPPH assay, is higher as SeNps are smaller. These authors evaluated SeNps of three different sizes and Na_2_SeO_3_ as ROS scavengers. In concordance with our findings, Na_2_SeO_3_ showed the poorest ROS scavenging activity when compared to SeNps. Matsuura et al. [64] evaluated the effect of L-Cys as a ROS scavenger when integrated to the surface of a 4.44 nm drug carrier L-serine (Ser)-modified polyamidoamine dendrimer. These authors concluded that L-Cys contributed to the ROS- and radical-scavenging efficacy when compared to the dendrimer without L-Cys. Significant antioxidant activity differences among SeNps and inorganic Se forms and positive controls has been previously reported [65,66].

Our results suggest that Se^0^Nps/L-Cys were more biocompatible than Na_2_SeO_3_ in RTgill-W1, RTS-11, and T-PHKM cells. Similar results were reported by Xu et al. [67] comparing cell viability after co-culturing SeNps or Na_2_SeO_3_ with human normal colon mucosal epithelial cells (NCM460). These authors reported a significant reduction of the viability of NCM460 cells by ≥0.39 μg/mL Na_2_SeO_3_ while the cell toxicity of *Lactobacillus casei* 393 strain biosynthesized SeNps was observed in the presence of 25 μg mL^−1^ Na_2_SeO_3_. A greater antioxidant activity of Se^0^Nps/L-Cys than Na_2_SeO_3_ has also been demonstrated in a cellular model using human umbilical vein endothelial cells (HUVEC) [30].

The pre-treatment of RTgill-W1, RTS-11, or T-PHKM cells with Se^0^Nps/L-Cys effectively reduced, exceeding Na_2_SeO_3_, the oxidative effect of H_2_O_2_ on the two cell lines and the primary cell culture assayed in the present work. Studies support that the exposition of a cell culture to 100 µM H_2_O_2_ causes cellular oxidative damage and/or OS [68]. According to Mou et al. [69], metabolic alterations in cells (melanocytes) by the oxidative effect of H_2_O_2_ directly influence the rate increase of cell apoptosis. In addition, the pre-treatment with 640 nM Se^0^Nps/L-Cys was a better attenuator of H_2_O_2_-induced oxidative damage than 640 nM Na_2_SeO_3_, improving the cell viability and reducing intracellular ROS concentration in the cells studied.

Our results, using RTgill-W1 and RTS-11 cells, suggest that Se^0^Nps/L-Cys could also contribute to alleviate the effect of oxidative environmental pollutants able to damage gill tissue of rainbow trout. According to Franco et al. [70] and Bopp et al. [71] the greater sensitivity of RTgill-W1 cells to ROS inducing toxins, when compared to some other cell types, could be related to a greater tendency for DNA fragmentation. In this sense, Ucar et al. [72] revealed insecticides, one of the most worldwide common environmental pollutants which negatively affect the health of aquatic organisms, including fish, produce higher genotoxicity and apoptosis in gill cells than in liver cells of rainbow trout due to oxidative damage [73]. Tkachenko et al. [74] assessed the effect of vaccination on the oxidative status of rainbow trout, showing that the activities of GPx, as well as glutathione reductase (GR), were significantly reduced in the muscles and gills of trout vaccinated against furunculosis suggesting that vaccination induced oxidative stress in these organs.

An increase of the activity of plasmatic lysozyme was observed in the rainbow trout receiving Se^0^Nps/L-Cys in their diet. The increase of plasma lysozyme levels in fish may be associated to an increased proliferation rate of phagocytic cells or to an increase in the number of lysosomes; therefore, assessing the activity of this enzyme seems to be an appropriate marker to evaluate the innate immune response in fish [75]. Kohshahi et al. [11] reported a significant increase of lysozyme activity when rainbow trout food was supplemented with chemically synthetized Se^0^Nps as compared to a dietary enrichment with Na_2_SeO_3_. Harsij et al. [76] also reported a significant increase of plasmatic lysozyme in rainbow trout administered synthetic Se^0^Nps combined with vitamins C and E. The use of Se^0^Nps as food supplement in Nile tilapia (*Oreochromis niloticus*) also significantly increased their plasma lysozyme activity when compared to the control group and to the group receiving Na_2_SeO_3_ supplementation [77].

Phagocytes produce respiratory bursts for the purpose of eliminating foreign pathogens during phagocytosis and have been widely used to evaluate the defense against pathogens. Superoxide anion along with hydroxyl radicals and nitric oxides are induced reactive oxygen species, which are related to enhancing microbial killing capacity of macrophages [78,79]. Data from the present study showed that rainbow trout fed with Se^0^Nps/L-Cys had higher respiratory burst activity (increase in the concentration cytoplasmatic ROS of blood leukocytes) on days 30 of the feeding trial when compared with the other groups. These results agreed with reports by Dawood et al. [80] and Xia et al. [81] who showed an increase in respiratory burst in blood phagocytes of *O. niloticus* and *Danio rerio*, respectively, fed for 8 weeks [82] and 9 days [83], respectively, with diets enriched with chemically synthesized Se^0^Nps.

All organisms have developed a variety of antioxidant defense systems to constantly suppress the production of ROS and remove them in cells of aerobic organisms [50]. Glutathione peroxidases (Gpxs) represent an important enzyme family, which protects living organisms from oxidative damage, catalyzing the reduction of H_2_O_2_ and organic hydroperoxides [83]. The Gpx activity in blood (plasma), liver, and muscle suggests that enriching the diet with Se^0^Nps/L-Cys would induce a better capacity of the antioxidant system to counteract the effect of ROS on the tissues of rainbow trout because it favors a larger Gpx activity, as already reported by Saffari et al. [54]. These authors reported that plasma Gpx was significantly higher in common carps (*Cyprinus carpio*) fed with Se^0^Nps than in fish treated with a basal diet (control) or a diet enriched with Na_2_SeO_3_. Naderi et al. [26] reported a significantly high Gpx activity in the hepatic tissue of rainbow trout receiving chemically synthetized Se^0^Nps when compared to the control animals. Khan et al. [50] indicated that the dietary administration of Se^0^Nps significantly increased Gpx activity in liver and muscle tissues of juvenile *Tor putitora* when compared to the control.

No relationship was observed between food supplemented with Se^0^Np, Se^0^Np/L-Cys, or Na_2_SeO_3_ and fish weight. This observation agrees with reports by Naderi et al. [26] who evaluated the effect of dietary supplementation with Se^0^Nps on SGR and other production parameters in *O. mykiss* under stress causing conditions. Nevertheless, Harsij et al. [76] reported a significant increase of the growth rate in juvenile rainbow trout chronically exposed to sublethal concentrations of ammonium and fed with food supplemented with a mixture of chemically synthetized Se^0^Nps and vitamins C and E when compared to the control (only ammonium). The authors postulated that the assayed mixture may have favored the synthesis of the selenoenzyme deiodinase, which is directly involved in the release of the growth hormone from the pituitary gland in vertebrates, including fish [84].

In the present study, the final condition factor (FCF) at the end of the assay, day 30, was better in rainbow trout receiving the Se^0^Nps/L-Cys supplemented diet. A FCF above 1.00 corresponds to a good health condition or well-being of fish and it correlates with an increase of important production parameters, such as fertility rate, which involves the production of high-quality gametes [85,86]. Our results suggest that supplementation of the diet with Se^0^Nps/L-Cys, when compared to Na_2_SeO_3_, may favor a better efficiency of rainbow trout accumulating energy reserves.

## 5. Conclusions

Supplementation of rainbow trout diet with Se^0^Nps/L-Cys had positive effect on fish innate immune response parameters, oxidative status, well-being, and growth. Se^0^Nps/L-Cys is a promising alternative for nutritional supplementation for rainbow trout with better performance than Na_2_SeO_3_, ease of implementation, and reduced environmental impact.

## Figures and Tables

**Table 1:** In vitro radical scavenging capacity of 500 µg mL^−1^ Se^0^Nps/L-Cys, Se^0^Nps, and Na_2_SeO_3_.

Antioxidant	DPPH IC_50_ (mg mL^−1^) ± SD	FRAP (TEAC mM) ± SD	TRAP (TEAC mM) ± SD
Se^0^Nps/L-Cys	1.96 ± 0.71	0.10 ± 0.03	0.19 ± 0.04
Se^0^Nps	2.53 ± 0.91	0.09 ± 0.01	0.15 ± 0.02
Na_2_SeO_3_	3.47 ± 0.49	0.06 ± 0,01	0.08 ± 0.01
Vit C	0.77 ± 0.08	0.26 ± 0.06	0.85 ± 0.01
Trolox	1.14 ± 0.06	n.a	n.a
NAC	1.42 ± 0.19	0.05 ± 0.02	0.16 ± 0.01

DPPH: radical scavenging 2,2′-diphenyl-1-picrylhydrazyl assay; FRAP: ferric reducing antioxidant power assay; TRAP: total radical-trapping antioxidant parameter assay; IC_50_: half-maximal inhibitory concentration. TEAC: Trolox equivalent antioxidant capacity; Vit C: vitamin C; NAC: N-acetylcysteine; n.a: not applicable.

**Table 2:** Effect of Se^0^Nps/L-Cys or Na_2_SeO_3_ on the cell viability of cell lines RTgill-W1, RTS-11, and T-PHKM.

Cells	Se^0^Nps/L-Cys (nM)			Na_2_SeO_3_ (nM)		
	160	320	640	160	320	640
RTgill-W1	95.64 ± 1.83	94.47 ± 2.22	92.66 ± 1.97	93.43 ± 2.35	92.05 ± 2.23	90.25 ± 1.67
RTS-11	96.39 ± 1.13	96.92 ± 1.45	95.67 ± 1.20	95.35 ± 1.65	95.05 ± 1.94	93.74 ± 2.21
T-PHKM	96.52 ± 0.43	96.05 ± 1.25	94.02 ± 1.13	96.13 ± 1.25	94.23 ± 1.30	93.33± 1.30

Results are expressed as percentage of viable cells when compared to control (cells not subjected to Se) assigned as 100%.

**Table 3:** Plasma lysozyme activity (in U mL^−1^) in rainbow trout fed with 5 mg kg^−1^ Se dry diet supplemented food for 30 days.

	Dietary Treatment			
Day	Control	Se^0^Nps	Se^0^Nps/L-Cys	Na_2_SeO_3_
0	36.37 ± 3.9	35.11 ± 4.1	35.25 ± 3.3	36.56 ± 3.8
15	37.14 ± 4.1	39.13 ± 4.7	41.47 ± 2.8	38.21 ± 3.6
30	37.53 ± 2.1	43.34 ± 2.6	46.40 ± 2.5	41.07 ± 3.3

One unit (U) of lysozyme activity corresponds to the amount of lysozyme that caused a decrease in absorbance of 0.001 min^−1^. Se^0^Nps: Non-functionalized biogenic Se nanoparticles, Se^0^Nps/L-Cys: L-cysteine functionalized biogenic Se nanoparticles. Data is given as mean ± SD; *n* = 15 in each sampling day per dietary treatment.

**Table 4:** ROS production by blood leukocytes, evaluated by NBT reduction into formazan, in rainbow trout. Fish were fed with 5 mg kg^−1^ Se dry diet supplemented food for 30 days.

		Dietary Treatment		
Day	Control	Se^0^Nps	Se^0^Nps/L-Cys	Na_2_SeO_3_
0	0.46 ± 0.014	0.44 ± 0.013	0.44 ± 0.11	0.45 ± 0.18
15	0.43 ± 0.017	0.49 ± 0.020	0.46 ± 0.14	0.44 ± 0.11
30	0.40 ± 0.021	0.45 ± 0.013	0.49 ± 0.02	0.41 ± 0.24

NBT: nitroblue tetrazolium, Se^0^Nps: Biogenic Se nanoparticles (non-functionalized), Se^0^Nps/L-Cys: L-cysteine functionalized biogenic Se nanoparticles; Data is given as mean ± SD; *n* = 15 in each sampling day per dietary treatment.

**Table 5:** Glutathione peroxidase (Gpx) activity in rainbow trout fed with 5 mg kg^−1^ Se dry diet supplemented food at day 30.

Gpx Activity	Dietary Treatment			
	Control	Se^0^Nps	Se^0^Nps/L-Cys	Na_2_SeO_3_
PlasmaLiver	257.36 ± 4.30	274.25 ± 5.86	279.39 ± 7.17	271.80 ± 8.47
	22.01 ± 2.06	28.31 ± 3.31	29.46 ± 3.71	26.67 ± 3.19
Muscle	25.98 ± 2.85	36.26 ± 3.64	40.06 ± 3.04	34.02 ± 1.83

Gpx: Glutathione peroxidase expressed in mU mg^−1^ protein. Se^0^Nps: Non-functionalized biogenic Se nanoparticles, Se^0^Nps/L-Cys: L-Cys functionalized biogenic Se nanoparticles. Data is given as mean ± SD; *n* = 15 per dietary treatment.

**Table 6:** Growth performance and survival rate of rainbow trout fed with 5 mg kg^−1^ Se dry diet supplemented food for 30 days.

Index	Dietary Treatment			
	Control	Se^0^Nps	Se^0^Nps/L-Cys	Na_2_SeO_3_
IW (g fish^−1^)	104.57 ± 4.69	102.26 ± 4.63	101.77 ± 4.63	103.11 ± 4.64
FW (g fish^−1^)	174.45 ± 3.86	178.14 ± 3.59	179.66 ± 2.71	175.76 ± 2.61
WG (g)	70.54 ± 6.63	76.28 ± 6.15	78.00 ± 4.25	72.69 ± 6.69
SGR (%)	1.72 ± 0.18	1.86 ± 0.17	1.89 ± 0.12	1.78 ± 0.21
ICF (%)	1.24 ± 0.11	1.26 ± 0.19	1.22 ± 0.12	1.23 ± 0.10
FCF (%)	1.27 ± 0.24	1.52 ± 0.26	1.68 ± 0.35	1.45 ± 0.23
Survival rate (%)	100	100	100	100

Se^0^Nps: Biogenic Se nanoparticles (non-functionalized), Se^0^Nps/L-Cys: L-cysteine functionalized biogenic Se nanoparticles; IW: initial weight; FW: final weight; WG: weight gain; SGR: specific growth rate; ICF: initial condition factor; FCF: final condition factor. Survival rate at the end of the assay (day 30); Data is given as mean ± SD; *n* = 15.

## Data Availability

Not applicable.
